# Application of native T1 map in characterization of acute myocardial infarction: can T1 distinguish between infarct area and area-at-risk?

**DOI:** 10.1186/1532-429X-18-S1-P96

**Published:** 2016-01-27

**Authors:** Nicola Galea, Marco Francone, Pierpaolo Palumbo, Laura De Luca, Luciano Agati, Carlo Catalano, Iacopo Carbone

**Affiliations:** 1 grid.7841.aDepartment of Radiological, Oncological and Pathological Sciences, Sapienza - University of Rome, Rome, Italy; 2 grid.7841.aDepartment of Cardiology, Sapienza - University of Rome, Rome, Italy

## Background

Combined assessment of edema and necrosis using conventional T2-weighted sequences and late gadolinium enhanced (LGE) imaging has been established as the reference standard for in-vivo assessment of myocardial damage in acute myocardial infarction (AMI). However the standard CMR protocol is time-consuming, not tolerated by patients in poor clinical conditions and needs of gadolinium administration. Moreover, T2 weighted images are not always of good image quality and are more prone to be affected by artifacts. Our purpose was to investigate the capability of native T1-mapping to differentiate with a quantitative approach the infarcted area, area-at-risk and the healthy myocardium.

## Methods

Twenty-seven consecutive patients performed CMR within the first 7 days following STEMI. CMR protocol included MOLLI, STIR T2w and cineMR sequences. LGE images were acquired after administration of a bolus of 0.2 mmol/Kg gadoterate meglumine (Gd-DOTA). MOLLI images were analyzed with a dedicated software (Cvi42, Circle) by placing four ROIs within necrotic areas (LGE area, excluding area of microvascular obstruction, MVO), area-at-risk (hyperintense area on T2 weighted images without LGE) and remote healthy myocardium on the same image. Acquisition time of each sequence was measured. Results are expressed on mean ± SD and compared with Student's t test.

## Results

Mean T1 native value of patients (age 59 ± 10 yrs) was 1357 ± 72 ms in necrotic area (LGE+/MVO-),1141 ± 57 ms in area-at-risk (STIR+/LGE-) and 961 ± 79 ms in remote myocardium (LGE-/STIR-). Significant differences were found in the comparison of T1 values between all regions (p < 0.01 for all). Infarct size was 25 ± 19% of left ventricular mass. Acquisition time of cineMR+mapping protocol was 22.7 ± 8.6 min, whereas standard CMR protocol was 46.4 ± 9.0 min (p < 0.01).

## Conclusions

Native T1 mapping may distinguish necrotic area, area-at-risk and healthy myocardium in reperfused AMI. It might assess myocardial injury in shorter time and without contrast injection compared to conventional CMR approach.

**Figure 1:**
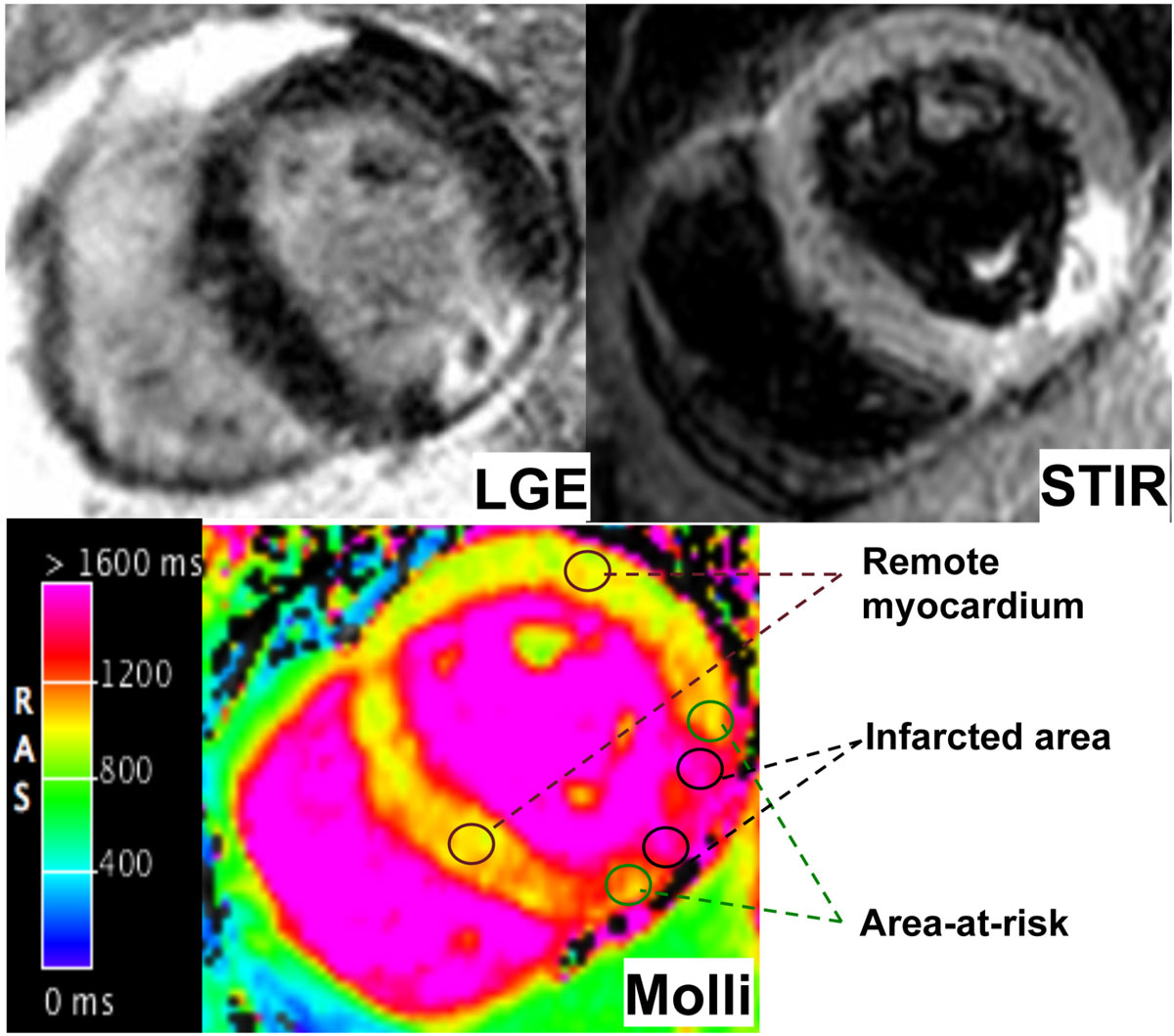
Figure 1

